# Hybridization and pre-zygotic reproductive barriers in *Plasmodium*

**DOI:** 10.1098/rspb.2014.3027

**Published:** 2015-05-07

**Authors:** Ricardo S. Ramiro, Shahid M. Khan, Blandine Franke-Fayard, Chris J. Janse, Darren J. Obbard, Sarah E. Reece

**Affiliations:** 1 Institute of Evolutionary Biology, University of Edinburgh, Ashworth Laboratories, West Mains Road, Edinburgh EH9 3JT, UK; 2 Centre for Immunity, Infection and Evolution, University of Edinburgh, Ashworth Laboratories, West Mains Road, Edinburgh EH9 3JT, UK; 3 Institute of Immunology and Infection Research, University of Edinburgh, Ashworth Laboratories, West Mains Road, Edinburgh EH9 3JT, UK; 4 Department of Parasitology, Leiden Malaria Research Group, LUMC, Albinusdreef 2, ZA Leiden 2333, The Netherlands

**Keywords:** malaria, hybridization, transmission, reproductive isolation, P230, P48/45

## Abstract

Sexual reproduction is an obligate step in the life cycle of many parasites, including the causative agents of malaria (*Plasmodium*). Mixed-species infections are common in nature and consequently, interactions between heterospecific gametes occur. Given the importance of managing gene flow across parasite populations, remarkably little is understood about how reproductive isolation between species is maintained. We use the rodent malaria parasites *P. berghei* and *P. yoelii* to investigate the ecology of mixed-species mating groups, identify proteins involved in pre-zygotic barriers, and examine their evolution. Specifically, we show that (i) hybridization occurs, but at low frequency; (ii) hybridization reaches high levels when female gametes lack the surface proteins P230 or P48/45, demonstrating that these proteins are key for pre-zygotic reproductive isolation; (iii) asymmetric reproductive interference occurs, where the fertility of *P. berghei* gametes is reduced in the presence of *P. yoelii* and (iv) as expected for gamete recognition proteins, strong positive selection acts on a region of P230 and P47 (P48/45 paralogue). P230 and P48/45 are leading candidates for interventions to block malaria transmission. Our results suggest that depending on the viability of hybrids, applying such interventions to populations where mixed-species infections occur could either facilitate or hinder malaria control.

## 1. Introduction

Interactions between species lie at the core of evolution because they can facilitate, or undermine, reproductive isolation and the process of speciation [1]. Mating interactions between heterospecifics can also shape geographical distributions of species via reproductive interference, the phenomenon in which the fitness of both (symmetric) or one (asymmetric) of the interacting species is reduced [2]. Reproductive isolation requires the evolution of barriers to genetic exchange between species and can act before (pre-zygotic) or after (post-zygotic) mating [3]. Pre-zygotic barriers include spatial or temporal segregation, behavioural isolation (e.g. through mate choice), gametic incompatibility and lack of gamete transfer/activation [3,4]. Post-zygotic barriers generally occur through hybrid sterility and inviability [3,4]. For *Plasmodium* (and related Apicomplexan) parasites, a single round of sexual reproduction is obligatory for transmission between hosts and parasites are hermaphroditic organisms that can self-fertilize and outcross. Developmentally arrested male and female sexual stages (gametocytes) are produced throughout infections in the vertebrate host and are taken up in the vector's blood meal. Once inside a blood meal, gametocytes have 30–60 min to differentiate into gametes and achieve fertilization [5]. However, the mechanisms responsible for the origin and maintenance of reproductive isolation are unknown.

Mixed-species infections of *Plasmodium* are common in humans (e.g. 12–65% in Thailand; [6]) and were present in approximately 28% of wild-caught rodent *Plasmodium* isolates [7]. Several lines of evidence suggest that gametocytes from co-infecting species co-transmit to vectors during blood feeding: (i) multiple *Plasmodium* species naturally infect the same host species, including humans (*P. falciparum*, *P. knowlesi*, *P. malariae*, *P. ovale* and *P. vivax*) and thicket rats (*P. berghei*, *P. chabaudi*, *P. vinckei* and *P. yoelii*) [6,8]. (ii) Co-infecting species concurrently produce gametocytes [9]. (iii) Mixed-species infections are found in wild captured *Anopheles* mosquitoes [10], and mosquitoes in the laboratory can simultaneously acquire and transmit multiple species [10,11]. (iv) The cues that stimulate gametogenesis are conserved across *Plasmodium* species (e.g. temperature drop > 5°C, xanthurenic acid [12,13]). Given the drive to develop interventions that block disease transmission by preventing mating [14], determining how heterospecific gametes interact is necessary.

Fertilization generally involves gamete attachment and recognition (potentially at the same time), followed by fusion [15]. In *Plasmodium*, the following proteins are required for conspecific gamete interactions during fertilization. HAP2/GCS1 is expressed at the surface of male, but not female gametes, and is required for fusion, but not attachment [16,17]. The proteins P230, P47 and P48/45 belong to the 6-cys multi-domain protein family and are expressed at the surface of gametocytes/gametes [18,19]. P47 (a paralogue of P48/45) is only expressed at the surface of female gametes and its deletion in *P. berghei* prevents viable male gametes from attaching to females [18,20]. P230 and P48/45 are important targets of transmission-blocking immunity [21] and are expressed at the surface of both male and female gametes [18,22]. Deletion of either P230 or P48/45 in *P. berghei* renders males infertile but has no apparent impact on female fertility [18,22]. However, the fertility of female gametes lacking P230 or P48/45 has only been investigated in mating crosses between conspecifics [18,22].

We investigated what, if anything, prevents gene flow between different *Plasmodium* species using two rodent malaria species, *P. berghei* and *P. yoelii,* as model systems (see ‘Supplementary methods’ for the rationale on using these species, electronic supplementary material). We determined that pre-zygotic barriers do exist, and that heterospecific mating occurs at a high rate when P230 or P48/45 is absent from the surface of female gametes. Therefore, our approach has identified proteins important for reproductive isolation, which is of particular relevance for taxa where hybridization is suspected (e.g. *Haemoproteus* [23,24]). We then examined the ecology of mixed-species mating groups and show that asymmetric reproductive interference occurs. Finally, because organisms with external fertilization often have fast-evolving gamete recognition proteins [25], we examined the rate of evolution of *p230*, *p48*/45 and the related gene, *p47*.

## 2. Material and methods

**Figure 1.**
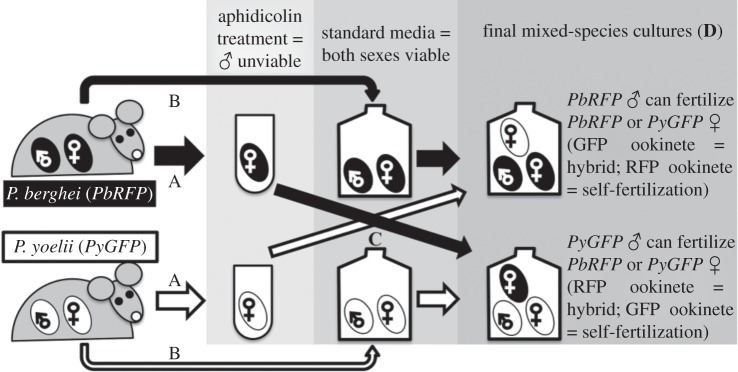
Experimental design for mixed-species mating cultures of intact parasites. Blood was taken from mice infected with *P. berghei* (*PbRFP*—black circles; RFP-expressing females/ookinetes) or *P. yoelii* (*PyGFP*—white circles; GFP-expressing females/ookinetes). Parasites were incubated in media that did (A) or did not (B) contain aphidicolin. Aphidicolin is an inhibitor of DNA polymerase-α that makes males unviable because male gametocytes, unlike females, have to replicate their DNA to produce gametes. Aphidicolin was washed off and parasites then were added to cultures containing heterospecifics (C) in vector mimicking media, which triggers gametogenesis and fertilization. Final cultures (D) contained fertile males from a single species (at a high enough density to ensure they were not limiting) and females from both species, expressing different fluorescent proteins.

We carried out two experiments taking advantage of the ability of *P. yoelii* or *P. berghei* to mate in culture. Compared with mosquito transmission experiments, our *in vitro* approach allowed us to more accurately standardize the conditions parasites experienced and simultaneously set up and assay a larger number of samples. The first experiment tested whether hybridization occurs between *P. yoelii* and *P. berghei,* in reciprocal crosses between males and females of each species (referred to as ‘intact’ parasites). The second experiment examined hybridization rates between *P. yoelii* males and *P. berghei* female gametes that could not produce either P230 or P48/45 (referred to as ‘knockout’ parasites). We used genetically modified reference lines of *P. berghei* and *P. yoelii* whose female gametes and ookinetes express different fluorescent proteins to distinguish between offspring resulting from conspecific and heterospecific fertilizations (see figure 1 for experimental design). The parasite lines used and their phenotypes with respect to the fertility of male/female gametes and expression of fluorescent proteins are shown in electronic supplementary material, table S1 and the numbers of infections contributing to each type of culture are shown in electronic supplementary material, table S2.

### (a) Hosts and parasites

We infected MF1 male mice, aged 8–12 weeks (Harlan-Olac, UK; or in-house supplier, University of Edinburgh), with either *P. yoelii* or *P. berghei* (as described in [26]). Two to four days before infection, we treated mice with phenylhydrazine (PHZ) to elevate gametocyte (gamete precursor stages) production [27]. For the first experiment, using intact parasites, we inoculated mice with either 10^7^ red blood cells (RBCs) parasitized with *P. berghei* lines *PbRFP* or *PbΔp47* (PHZ: 125 mg kg^−1^, day −2 post-infection (PI)) or 10^8^ RBCs parasitized with *P. yoelii* lines *PyGFP* or *Py*17X (PHZ: 60 mg kg^−1^, day −3PI). For the second experiment, using knockout parasites, we inoculated mice with 10^7^ RBCs parasitized with either *P. berghei* lines *PbWT*, *PbΔp230, PbΔp48/45* or with *P. yoelii* line *PyWT* (60 mg kg^−1^ PHZ, day −4 PI). These parasite and PHZ dose combinations ensured high gametocyte densities within a few days of infection, so that the presence of transmission-blocking immune factors was minimized.

### (b) Mating cultures

We performed all of the experiments using the conditions described in [26]. Briefly, we harvested gametocytes (on day 3 or 4 post-infection), from mice infected either with *P. berghei* or *P. yoelii*, and incubated them in RPMI (Roswell Park Memorial Institute) media with 10% calf serum at pH 8 and 21°C. This mimics the vector environment, immediately triggering gametogenesis, fertilization and ookinete development [26]. *P. yoelii* is thought to transmit at 24°C but preliminary work revealed that culturing at 21°C does not significantly affect fertilization success. We counted the number of females and exflagellating males present in each infection (as described in [26]) to determine the densities of males and females of each species in the mating cultures. Each infected mouse contributed parasites to only one mixed-species culture and to one culture of each the controls (see below). This maximizes statistical power while avoiding pseudo-replication [26].

To measure both con- and heterospecific fertilization success, we assayed ookinete numbers 18–20 h after fertilization (when ookinetes have developed). To do this, we examined 10 μl of each culture in a Neubauer haemocytometer and counted ookinetes using a fluorescence microscope. Zygotes resulting from heterospecific matings can develop into ookinetes because males make little contribution to zygote-to-ookinete development. Ookinetes develop via translationally repressed proteins, whose mRNAs are present in females before mating [28]. Indeed, male gene expression has not been observed until the ookinete-to-oocyst transition [29]. We used ookinetes to assay fertilization success because, compared with zygotes, the distinct crescent shape of ookinetes means they can be more accurately distinguished from unfertilized females. We distinguished ookinetes resulting from conspecific and heterospecific fertilizations by the fluorescent protein that they expressed (figure 1; electronic supplementary material, table S1).

### (c) Hybridization between ‘intact’ *Plasmodium berghei* and *Plasmodium yoelii*

We tested whether hybridization occurs between ‘intact’ (i.e. non knockout) *P. berghei* and *P. yoelii* gametes by mixing both species together in mating cultures after making the males of one species infertile. Therefore, in one set of cultures the *P. berghei* males were infertile and so, hybrid ookinetes could only be produced by matings between *P. berghei* females and *P. yoelii* males and would express red fluorescent protein (RFP). Conversely, conspecific matings would give rise to ookinetes expressing green fluorescent protein (GFP). In the second set, the *P. yoelii* males were infertile, and so hybrid ookinetes (GFP) could only occur when *P. berghei* males mated *P. yoelii* females (conspecific ookinetes: RFP).

To make males infertile we used aphidocolin, which stops male gametogenesis but leaves females unaffected. We incubated 15 μl of *PbRFP-* or *PyGFP*-infected blood (for 12 min) in 1 ml RPMI with 5 × 10^−4^ M aphidicolin (Sigma-Aldrich, UK) [22]. We then washed the aphidicolin by centrifuging (12000 r.p.m., 5 s) and replacing the supernatant with new RPMI (without aphidicolin). During the time that parasites were undergoing aphidicolin treatment, we collected parasites from all other *PbRFP* or *PyGFP* infections and added 60 μl infected blood to 4 ml RPMI. We then combined the cultures of infertile males plus fertile females with cultures of the other parasite species that contained viable males and females (final culture volume: 5 ml). These steps are illustrated in figure 1. We used different volumes of blood (15 μl and 60 μl) to ensure a high ratio of viable males relative to females, minimizing the possibility of male limitation constraining fertilization of either con- or heterospecific females.

#### (i) Control cultures

Several types of control cultures were required to validate that (i) conspecific mating occurs within the *PbRFP* and *PyGFP* lines; (ii) aphidicolin treatment did not adversely affect *PyGFP* and *PbRFP* females; (iii) aphidicolin treatment blocked male fertility of *PyGFP* and *PbRFP*. We verified these assumptions by (i) independently culturing *PbRFP*- or *PyGFP*-infected blood and observing ookinetes; (ii) inactivating *PbRFP* or *PyGFP* males with aphidicolin and mixing them with the conspecifics *P. berghei Δp47* (only males are fertile) and *P. yoelii* 17X wild-type (ookinetes are wild-type) and observing fluorescent ookinetes; (iii) inactivating *PbRFP* or *PyGFP* males with aphidicolin and not observing ookinetes. See electronic supplementary material, table S2 for the number of replicates and the ookinete densities produced, per control type. A full description of these results is given in electronic supplementary material, ‘Supplementary results’.

### (d) Hybridization between P230 and P48/45 ‘knockouts' and *Plasmodium yoelii*

To test whether P230 and/or P48/45 mediate species recognition, we set up mating crosses between a wild-type line of *P. yoelii* (*PyWT*) and *P. berghei* lines that constitutively express GFP and lack either *p230* (*PbΔp230*) or *p48/45* (*PbΔp48/45*; electronic supplementary material, table S1). The deletion of P230 or P48/45 renders males unviable, so aphidocolin treatment was not required. Thus, we simply mixed 10 μl of *PyWT*-infected blood (males and females are viable) with 10 μl of *PbΔp230* or *PbΔp48/45* (only females are viable) in 1 ml cultures. We then assayed the densities of GFP ookinetes (mating between *P. yoelii* males and *P. berghei* females) or wild-type ookinetes (*P. yoelii* self-fertilizations).

#### (i) Control cultures

We set up control cultures to verify that (i) *PyWT* produces ookinetes but *PbΔp230* and *PbΔp48/45* do not and (ii) females from *PbΔp230* and *PbΔp48/45* can be fertilized by conspecific males. We verified these assumptions by (i) culturing each line alone and observing ookinetes in *PyWT* but not *PbΔp230* or *PbΔp48/45* cultures and (ii) mixing *P. berghei* wild-type with *PbΔp230* or *PbΔp48/45* and observing ookinetes. See electronic supplementary material, table S2 for the number of replicates and the ookinete densities produced, per control type. A full description of these results is given in electronic supplementary material, ‘Supplementary results’.

### (e) Data analysis

*Plasmodium berghei* and *P. yoelii* produce different numbers of gametocytes during infections, so the numbers of heterospecific and conspecific females differed in mixed-species cultures. However, because all mixed-species cultures contain fertile males from only one species, if parasites mated randomly, the proportion of hybrid ookinetes would be equal to the proportion of females that are heterospecific. Therefore, we term the proportion of heterospecific females as ‘expected hybridization*’* (under random mating) and the proportion of hybrid ookinetes as ‘observed hybridization’. In the majority of our analyses of the mixed-species cultures, we compare expected with observed hybridization because we are testing for deviations from random mating. All analyses were performed in R v. 2.14.0 (http://www.r-project.org/) and consisted of generalized-linear and linear mixed-effects models and *t*-tests. This depended on the distribution of the data, the need to account for random effects and sample sizes. Non-parametric Wilcoxon tests were used when the assumptions of normality could not be met by data transformation. Further details on data analysis of Results sections (§§3(b,d)) are given in electronic supplementary material, ‘Supplementary methods’.

### (f) Molecular evolution

We determined the DNA sequence of *p230*, *p48/45* and *p47* using previously collected genomic DNA [7], spanning 58 genotypes from field isolates of the four rodent malaria species (*P. berghei*, *P. chabaudi*, *P. vinckei* and *P. yoelii*; see electronic supplementary material, table S3 for primers and PCR cycling conditions). We sequenced the entire *p47* and *p48/45* loci. However, as *p230* is a large locus (approx. 8.3 kb), we examined two regions of this locus thought to be fast- or slow-evolving [18] (region I ranges from 2242 to 3466 bp and region II from 6741 to 7832 bp; reference: *P. berghei* at PlasmoDB, see http://plasmodb.org). We investigated the female-specific surface protein P47 because it plays a role in male recognition of female gametes and it has been suggested that it directly interacts with P230 and/or P48/45 during gamete recognition and attachment [18]. We refer to *p230*, *p47* and *p48/45* collectively as ‘mating’ loci (GenBank: KP849590–KP849808). Moreover, for some of the analysis, we included a set of 11 ‘control’, house-keeping loci (GenBank: JX904678–JX905153, JX984464–JX984513; [7]).

After aligning the sequences and testing for recombination, we computed a variety of standard population-genetic summary statistics: (i) counts of fixed differences at non-synonymous and synonymous sites (*D*_N_, *D*_S_) and polymorphisms (*P*_N_, *P*_S_); (ii) divergence ratios (substitutions per site: *K*_A_, *K*_S_, *K*_A_*/K*_S_) and nucleotide diversity (*π*_A_, *π*_S_, *π*_A_/*π*_S_) [30]; (iii) Tajima's *D* [31]; (iv) single-locus McDonald–Kreitman (MK) tests [32]; (v) multi-locus MK [33] and Hudson–Kreitman–Aguade tests (HKA, [34]). Moreover, we used codon evolution models to test whether the strength of selection varied along each locus [35]. Further details on each type of analysis are given in electronic supplementary material, ‘Supplementary methods’.

## 3. Results

### (a) Hybridization can occur between *Plasmodium berghei* and *Plasmodium yoelii*

**Figure 2.**
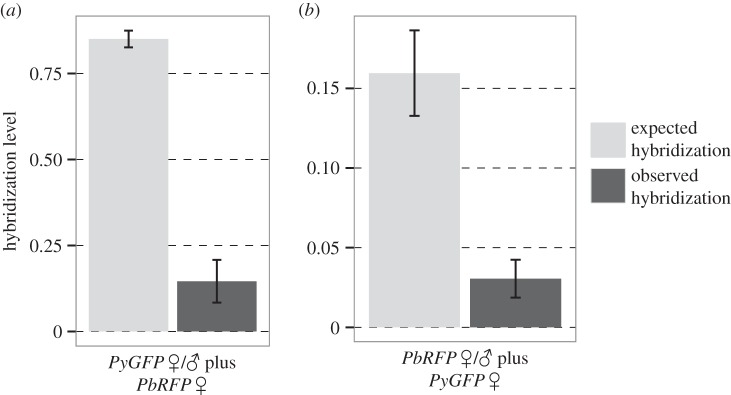
Hybridization occurs between *P. berghei* and *P. yoelii. Expected hybridization,* under random mating (i.e. proportion of heterospecific female gametes; light bars), and *observed hybridization* (i.e. proportion of hybrid ookinetes; dark bars). Mean ± s.e. is shown for matings between (*a*) *P. yoelii* (*PyGFP*) males and *P. berghei* (*PbRFP*) or *PyGFP* females and (*b*) *PbRFP* males and *PyGFP* or *PbRFP* females.

When we tested whether hybridization occurs between ‘intact’ lines of *P. berghei* and *P. yoelii*, we obtained hybrid ookinetes in 60% of the crosses between male *PyGFP* and female *PbRFP* (as identified by RFP-positive ookinetes) and in 40% of the crosses between male *PbRFP* and female *PyGFP* (GFP-positive ookinetes). However, the proportion of hybrid ookinetes *(observed hybridization*) was much lower than the proportion of heterospecific females (*expected hybridization*; figure 2). Specifically, in the cross between *PyGFP* males and *PbRFP* females, *expected hybridization* was on average 0.85 ± 0.02 (±s.e.) but *observed hybridization* was 0.15 ± 0.06 (Wilcoxon; *V* = 0; *p* < 0.0001; d.f. = 14). In the cross between *PbRFP* males and *PyGFP* females, *expected hybridization* was 0.16 ± 0.03 and *observed hybridization* was 0.03 ± 0.01 (Wilcoxon; *V* = 5; *p* < 0.0001; d.f. = 19; see electronic supplementary material, table S2 for ookinete densities). While this demonstrates that hybridization can occur between different species of malaria parasites, there is clearly strong preference for mating between conspecifics suggesting that pre-zygotic reproductive barriers operate during mating.

### (b) Reproductive interference

**Figure 3.**
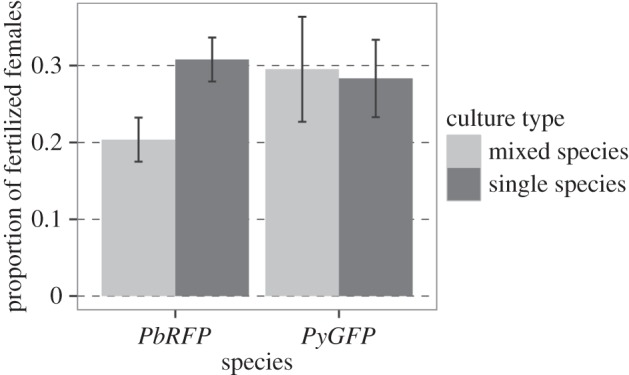
Asymmetric reproductive interference between *P. berghei* and *P. yoelii*. The proportion of conspecific females (mean±s.e.) that were fertilized in single- (dark bars) and mixed-species (light bars) cultures for *P. berghei* (*PbRFP*; left) and *P. yoelii* (*PyGFP*; right). Fertilization success is significantly reduced for *P. berghei*, but not *P. yoelii*, in mixed-species mating groups.

We used the data from the experiment above to test whether reproductive interference occurs. For *P. berghei,* self-fertilizations (i.e. the proportion of conspecific fertilized females) are reduced in the presence of *P. yoelii* (likelihood ratio test, 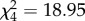, *p* < 0.001, single species cultures: 0.31 ± 0.03; mixed species: 0.20 ± 0.03). By contrast, the presence of *P. berghei* had no significant effect on *P. yoelii* self-fertilizations (LRT 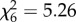; *p* = 0.51, single species: 0.28 ± 0.05). Therefore, asymmetric reproductive interference can occur and in particular, it reduces conspecific mating rates for *P. berghei* by approximately 30% (figure 3).

### (c) P230 and P48/45 are involved in pre-zygotic reproductive barriers

**Figure 4.**
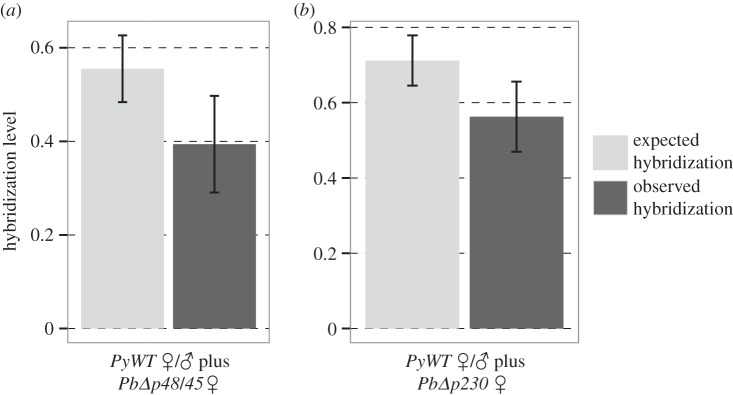
Hybridization between *P. yoelii* males and *P. berghei* females occurs at higher rates when *P. berghei* females lack P48/45 or P230. *Expected hybridization,* under random mating (i.e. proportion of heterospecific female gametes; light bars), and *observed hybridization* (i.e. proportion of hybrid ookinetes; dark bars). Mean ± s.e. is shown for matings between (*a*) *P. yoelii* (*PyWT*) males and *PbΔp48/45* or *PyWT* females; and (*b*) *PyWT* males and *PbΔp230* or *PyWT* females.

Next, we tested whether P230 and P48/45 proteins influence the levels of observed hybridization. We obtained hybrid ookinetes in crosses between male *PyWT* and females from *PbΔp230* (approx. 80% of the cultures) and *PbΔp48/45* (approx. 90% of the cultures). In contrast to the crosses between intact parasites, *observed hybridization* approached the levels of *expected hybridization* (cf. figures 2 and 4). Specifically, while the mean difference between *expected* and *observed hybridization* was 0.70 (95% CI: 0.58–0.83; assuming a normal distribution) for crosses between ‘intact’ *P. yoelii* males and *P. berghei* females, this difference was approximately five times smaller for crosses between *P. yoelii* males and *PbΔp230* females (0.14; 95% CI: 0.016–0.28) or *PbΔp48/45* females (0.16; 95% CI: −0.04–0.36; see electronic supplementary material, table S2 for ookinete densities). Moreover, while there was a significant difference between *expected* (0.71 ± 0.07) and *observed hybridization* (0.56 ± 0.09) for the cross between *PyWT* males and *PbΔp230* females (*t* = −2.49, d.f. = 10, *p* = 0.032), this was not the case for the cross between *PyWT* males and *PbΔp48/45* females (*t* = −1.75, d.f. = 13, *p* = 0.104; *observed hybridization*: 0.39 ± 0.10; figure 4), suggesting that *PyWT* males randomly mate with conspecific and *PbΔp48/45* females, but not *PbΔp230* females (we address this issue in the next section). These results indicate that P230 and P48/45, at the surface of female gametes, are key for pre-zygotic reproductive isolation.

### (d) Mating is non-random in the absence of P230 and P48/45

**Figure 5.**
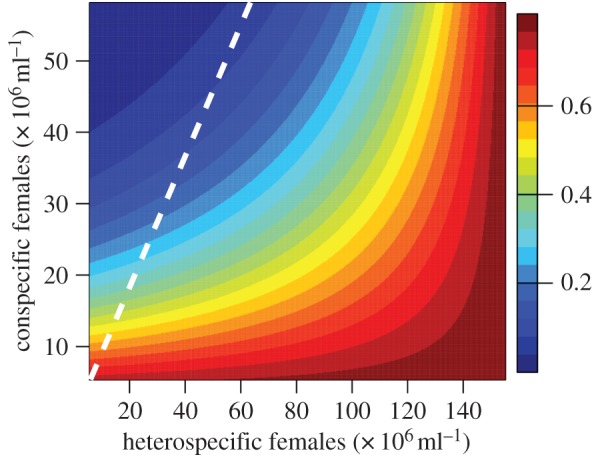
Hybridization rates correlate with the interaction between the densities of con- and heterospecific females. The proportion of matings that produce hybrids (red, high; blue, low) follows a nonlinear pattern with respect to the densities of con- (*y*-axis) and heterospecific females (*x*-axis) available in culture. When the densities of con- and heterospecific females are equal (white line), random mating would result in 50% of matings producing hybrids. The yellow band (50% hybrids) mostly sits to the right of the white line. This suggests that fewer hybrids are observed than expected under random mating for most combinations of con- and heterospecific female densities and so P230 and P48/45 are not the only pre-zygotic barriers.

The number of con- and heterospecific female gametes was variable in cultures of *P. yoelii* males plus *PbΔp230* or *PbΔp48/45* females. This enabled us to examine whether *P. yoelii* males randomly mate with conspecific and *PbΔp48/45* or *PbΔp230* females. Our analysis showed that *P. yoelii* males do not have a significant preference for *PbΔp48/45* or *PbΔp230* females (*F*_1,20_ = 0.813; *p* = 0.378). However, *observed hybridization* was significantly affected by the interaction between the densities of con- and heterospecific females available (*F*_1,21_ = 10.199, *p* = 0.004). To visualize this interaction, we generated a matrix of values for the densities of con- and heterospecific females (within the observed ranges). We then inputted this matrix into the minimal generalized-linear model (see electronic supplementary material, ‘Supplementary methods’) to predict how *observed hybridization* correlates with the density of con- and heterospecific females (figure 5). Unsurprisingly, *observed hybridization* rises as the density of heterospecific females increases and decreases when the density of conspecific females increases. However, these patterns are nonlinear; when the density of heterospecific females is low, the level of *observed hybridization* is dominated by the density of conspecific females (figure 5, colours change vertically), but at high densities of heterospecific females (greater than 120 × 10^6^ ml), *observed hybridization* becomes independent of the density of conspecific females (figure 5, colours change horizontally). The key point is that random mating is only predicted at very low densities of both con- and heterospecific females or at very high densities of heterospecific females.

### (e) P230, P47 and P48/45 are under positive selection

Since the surface proteins P230 and P48/45 play a role in conspecific gamete recognition [18,22] and mediate mating between heterospecific gametes (this study), we investigated the evolution of *p230*, *p48/45* and *p47* (‘mating’ loci) and identified domains under selection. In our analyses, single gene MK tests were significant only for region I of *p230*, with *α* varying between 0.7 and 0.85 (*α* is the estimated proportion of non-synonymous substitutions owing to positive selection [36]; electronic supplementary material, table S4). However, the greater statistical power of the multi-locus MK tests suggests that the ‘mating’ loci experience a higher rate of adaptive change (i.e. non-synonymous divergence) than the ‘control’ loci (table 1; see electronic supplementary material, tables S5 and S6, for the full multi-locus MK results). This is probably driven by region I of *p230* and *p47*, which are the only loci that better fit a model where *α* varies between ‘control’ and ‘mating’ loci (when each locus is separately tested; table 1 and tables S5 and S6). Moreover, for *p230* (region I) and *p47*, *α* is substantially higher than for the remaining loci (*α* varies between 0.7–0.86 and 0.52–0.82 for region I of *p230* and *p47*, respectively; electronic supplementary material, tables S5 and S6). On the other hand, the HKA test did not detect significant differences in polymorphism between mating and control loci (data not shown), for any of the comparisons tested. This suggests an absence of either long-term balancing selection or recent selective sweeps and is in agreement with the lack of significance of single-locus Tajima's *D* ([37];electronic supplementary material, table S4).

**Table 1.** The ‘mating’ loci are under positive selection. *α* is shown for the ‘mating’ loci individually or as a group (All) and for a group of 11 ‘control’ loci [7]. *α* was estimated using a multi-locus MK test [33]. *α* values in bold indicate loci for which the best-fitting model allowed *α* to vary between ‘mating’ and ‘control’ loci, which indicated that the ‘mating’ loci are adaptively evolving faster than the ‘control’. Data in this table were obtained using polymorphism counts for *P. chabaudi chabaudi* clones, but qualitatively similar results are obtained for the subspecies *P. y. yoelii* or *P. vinckei petteri* and at the species level (electronic supplementary material, tables S5 and S6).

	locus tested				
	All	*p230* region I	*p230* region II	*p47*	*p48/45*
*α*_mating_	**0.75**	**0.86**	0.67	**0.84**	0.45
*α*_control_	**0.37**	**0.47**	0.47	**0.39**	0.47

Finally, using codon evolution models ([35]; see electronic supplementary material, ‘Supplementary methods’), we detected positive selection across all mating loci, with estimated *K*_A_*/K*_S_ always above 2.3 for the most strongly selected class of codons (see electronic supplementary material, table S7 for details on statistical significance, *K*_A_*/K*_S_ values and % of selected codons). Moreover, we identified 65 candidate codons as being positively selected (across all loci) and therefore greatly expand on the 15 codons previously identified in [18] (electronic supplementary material, table S8). Taken together, these analyses suggest that the ‘mating loci’ (particularly *p47* and region I of *p230*) are evolving under positive selection, but there is no evidence for balancing selection or recent selective sweeps.

## 4. Discussion

We combined *in vitro* fertilization experiments with molecular evolution tools to examine the mating biology of *Plasmodium* species. We show that *P. berghei* and *P. yoelii* gametes can hybridize and that asymmetric reproductive interference occurs in mixed-species mating groups. Hybridization rates significantly increase when either of the proteins P230 or P48/45 is absent from the surface of *P. berghei* female gametes, suggesting an unexpected role for these proteins in species recognition during fertilization. However, the absence of these proteins does not lead to random mating between con- and heterospecifics, indicating that other, yet unidentified, factors also contribute to pre-zygotic barriers. Finally, we reveal strong positive selection on a region of P230 and on the female surface protein P47 and suggest specific codons in these genes that experience strong selection.

### (a) Hybridization and introgression

Recent research shows that hybridization occurs more frequently than previously thought [38], and that introgression can have important evolutionary consequences for diverse organisms, including the parasites *Schistosoma* and *Leishmania* (hybridization extends the vector-species range [39,40]). We observed low levels of hybridization between wild-type *P. yoelii* and *P. berghei* gametes (figure 2). To our knowledge, this is the first time that hybridization has been demonstrated for species of the *Plasmodium* genus (although hybridization has been reported in the related *Haemoproteus* genus [23]). Whether hybrids are able to complete the life cycle is not clear. We investigated this by carrying out a small number of mosquito feeds on mice co-infected with *P. yoelii* wild-type and GFP-labelled *P. berghei* lines lacking P230 or P48/45. We observed hybrid oocysts (GFP) of abnormally small size (without sporozoites) in mixed, but not in single-species infections (data not shown). This suggests that hybrids may often fail to proceed further than the early oocyst stage and fits with the failure of earlier attempts to produce crosses of these species *in vivo* [8]. However, experimentally assessing the viability and evolutionary impact of hybrids is very difficult because the number and genetic diversity of the parasites circulating in natural environments is much higher than what can be experimentally tested. Furthermore, introgression can be important at the population level even when the probability of individual hybrids completing the life cycle is extremely low. In this case, examining individual parasites will only very rarely reveal a fit hybrid. Thus, evidence for hybrid viability is better obtained from genome sequence data, as has been the case for schistosomes [39].

Interestingly, we also observe that, in the absence of P230 or P48/45, the frequency of hybridization changes nonlinearly with the interaction between the densities of con- and heterospecific females. Moreover, the frequency of hybridization approaches random mating very rarely. This is the case at very low densities of both con- and heterospecific females. Low gametocyte densities are the norm in natural infections [41], suggesting that hybridization may be more common than we observed. In this case, parasites could reduce chances of hybridization (assuming it is costly for fitness) by increasing the number of circulating gametocytes.

### (b) Reproductive interference

We demonstrate that asymmetric reproductive interference occurs in *Plasmodium,* in which the self-fertilization success of *P. berghei,* but not *P. yoelii*, is reduced in the presence of heterospecifics (figure 3). Similar results have been obtained by Paul *et al.* [42] and Valkiunas *et al.* [24] for avian *Plasmodium* and *Haemoproteus*, respectively. There are several mechanisms that could underpin this phenomenon, including (i) host immune factors produced in *P. yoelii* infections that act in the blood meal (e.g. [21,26]) could have more severe effects on *P. berghei* than on *P. yoelii*; (ii) if male gametes use chemotaxis to locate females, *P. yoelii* males may be better able to distinguish between con- and heterospecific signals than *P. berghei* males. (iii) Direct, chemically mediated, antagonistic interactions between gametes of different species (allelopathy) could also occur. Paul *et al.* [42] suggest that allelopathy occurs based on the observation that asymmetric reproductive interference among avian malaria parasites is independent of immunity. Whatever the reproductive interference mechanism, understanding whether reproductive interference occurs in the wild is important because it can be a determinant of epidemiological dynamics and geographical distributions of the interacting species [2].

### (c) The role of P230 and P48/45 in pre-zygotic isolation

We show that the absence of proteins P230 and P48/45 from the surface of female *P. berghei* gametes markedly increases hybridization with *P. yoelii* males (figures 2 and 4), suggesting that these proteins are involved in species recognition. An important role for these proteins in females was unexpected because all previous work has focused on their essential role for male fertility. While P230 and P48/45 are key for the maintenance of pre-zygotic barriers, it is unclear if P230 and P48/45 directly mediate species recognition, or whether P230 and P48/45 underpin the functionality of recognition proteins (e.g. P230 and P48/45 may ensure that recognition proteins are correctly localized). Importantly, our statistical model predicts that mating is generally non-random, despite the absence of P230 or P48/45, suggesting that other factors are also involved in species recognition/attachment (figure 5, §3d). Potential candidates include other male/female surface proteins, such as P47 or LAP/CCp-family members, which are known to interact with P230 and P48/45 [18,43]. It is also possible that species-specific chemotactic signals influence encounters between con- and heterospecific gametes.

While further work is required to determine if P230 or P48/45 or both are involved in mediating gamete recognition, there are several reasons to suspect that P230 plays a dominant role in pre-zygotic isolation, by mediating gamete recognition. First, P230 and P48/45 are expressed at the surface of male and female gametes [44,45] and form a complex, anchored to the gamete surface by P48/45 [46,47]. In *P. falciparum,* deletion of P48/45 prevents P230 expression at the gamete surface [47], but not vice-versa. Second, mating can occur in the absence of P230 and/or P48/45 from the surface of female gametes (§3 and [18]), suggesting that female recognition is not essential for fertilization to occur. Similar observations have been made for organisms as divergent as humans and hamsters [48]. Third, positive selection is commonly found in gamete recognition proteins across taxa and we find that region I of *p230*, but not *p48/45*, is fast evolving relative to the control loci. Fourth, the protein structure of *P. falciparum* P230 indicates that domain IV (defined in [19]) is an external domain available for molecular interactions—a domain where we identify eight fast-evolving codons and for which several non-synonymous polymorphisms have been identified in *P. falciparum* [19].

### (d) Molecular evolution of *p*230, *p*48/45 and *p*47

Our results provide evidence of adaptive evolution for the proteins involved in fertilization in *Plasmodium*, particularly for region I of *p230* and for *p47* (table 1). These results are in agreement with work on other taxa showing that genes involved in gamete recognition are fast-evolving [25] and provide further support for their role in gamete recognition. However, more work is needed to identify the ecological factors driving this fast evolution. In metazoans, sexual conflict/selection (e.g. polyspermy, assortative mating) or reinforcement are often thought to be the key forces driving this fast evolution [25]. While sexual conflict has not been studied in *Plasmodium*, natural transmission-blocking immunity targets P230 and P48/45, leading to reduced transmission success (e.g. [21]). Thus, natural antibodies could provide a selective pressure for driving the evolution of these proteins [49]. If P230 and/or P48/45 both contribute to reproductive isolation and are under divergent selection owing to immunity, this selective pressure could contribute towards non-random mating [50]. Such a pleiotropic effect cannot be broken down by recombination and so, could facilitate speciation in the presence of gene flow [51]. If this is the case, immunity against P230 or P48/45 could accelerate the rate at which *Plasmodium* lineages diverge. By contrast, no adaptive immune responses have been detected against P47 [20]. However, it may be coevolving with the mosquito immune response [52].

### (e) Conclusions and implications

Our results illustrate that considering the molecular and organismal interactions within an ecological context provides a broader understanding of the mating biology of parasites. While care should be taken in extrapolating from model systems to human parasites, the implications may have medical relevance. Hybridization may have complex consequences for the success of transmission-blocking interventions directed against P230 and P48/45. Both natural and vaccine-induced antibodies can greatly reduce transmission by complement-dependent or independent processes [21,53–55]. However, while complement-dependent processes lead to gamete lysis, complement-independent processes may only mask/inactivate P230 [53–55]. Thus, antibodies that induce complement-independent processes may simply interfere with gamete recognition/attachment, allowing fertilization to proceed. Because such antibodies will be species-specific, females of the target species could be fertilized by heterospecific males in blood meals from hosts with mixed-species infections. In this case, enhancing the production of antibodies to P230 (e.g. by vaccination) could facilitate hybridization. If hybrids are viable, introgression could facilitate the spread of medically unfavourable alleles (e.g. virulence determinants, drug resistance). However, if hybrids are not viable, facilitating hybridization may bring unexpected benefits by reducing transmission of both the target and the non-target species. Furthermore, given that specific regions of the ‘mating’ loci are fast-evolving, transmission-blocking vaccines should avoid targeting these epitopes in order to delay the emergence of vaccine-escape mutants, especially if selection pressures resulting from mate recognition and immunity target similar protein regions. Finally, understanding the molecular interactions responsible for reproductive interference could provide novel targets for transmission-blocking interventions.

## Supplementary Material

Text S1

## Supplementary Material

Table S3

## Supplementary Material

Dataset S1

## Data accessibility

Data for figures 2–5 is available as electronic supplementary material and DNA sequences are available at GenBank (KP849590–KP849808).
