# Supplementary material for: Hybridization and pre-zygotic reproductive barriers in Plasmodium
Source: Proc Biol Sci. 2015 May 7;282(1806):20143027. doi: 10.1098/rspb.2014.3027 (PMC4426616; doi:10.1098/rspb.2014.3027)
Supplement: Text S1 [file rspb20143027supp1.docx]

### ELECTRONIC SUPPLEMENTARY MATERIAL FOR:

## Hybridisation and pre-zygotic reproductive barriers in *Plasmodium*

Ricardo S. Ramiro, Shahid M. Khan, Blandine Franke-Fayard, Chris J. Janse, Darren J. Obbard, Sarah E. Reece

### CONTENTS

- Supplementary Methods (p. 1-8), including Tables S1-2 (p. 2-3), S3 (p. 7)
- Supplementary Results (p. 9-14), including Tables S5-8 (p. 11-14)
- Supplementary Legend for Table S4 (p. 15)
- Supplementary References (p. 15-16)

### SUPPLEMENTARY METHODS

### Model system

*P. berghei* and *P. yoelii* are excellent model systems for several reasons: (*a*) rodent malarias, especially *P. berghei*, are highly amenable for the study of sexual reproduction in *Plasmodium* [1] and other organisms [2]; (*b*) fluorescent reporter lines are available for both *P. berghei* and *P. yoelii* and mutant lines defective in expression of gamete surface proteins are available for *P. berghei* [3-5]; (*c*) *P. berghei* and *P. yoelii* are the closest related pair of rodent *Plasmodium* species and we recently estimated their divergence at 1.2-5.9 million years ago (Mya; similar to the *P. falciparum*-*P. reichenowi* divergence [6, 7]). However, a more recent estimate points to a the substantially older divergence time of 6.7-13.4 Mya [8]; (*d*) Both *P. berghei* and *P. yoelii* readily undergo transmission to mosquitos at 21 °C [9]; (*e*) the geographic distributions of *P. berghei* and *P. yoelii* are thought to overlap and *P. berghei* isolates, collected in Katanga (K713, NK65), also contained *P. yoelii* [10-12]; and (*f*) *P. yoelii* is the parasite most often found in mixed infections [6, 12].

**Table S1. Parasite lines used and their phenotypes with respect to the fertility of male and female gametes and the expression of fluorescent proteins.**

| **species** | **line** | **alias** | **experiment** | **phenotype** | **Reference** |
| --- | --- | --- | --- | --- | --- |
| *P. berghei* | ANKA | *PbRFP* | Intact parasites | RFP females and GFP males; RFP ookinetes; fertile gametes | [13] |
|  |  | *PbWT* | Knockout parasites | wild-type; fertile gametes |  |
|  |  | *PbΔp230* | Knockout parasites | P230 knock-out; constitutive GFP; infertile male gametes | [5] |
|  |  | *PbΔp47* | Intact parasites | P47 knock-out; constitutive GFP; infertile female gametes | [5] |
|  |  | *PbΔp48/45* | Knockout parasites | P48/45 knock-out; constitutive GFP; infertile male gametes | [4] |
| *P. yoelii yoelii* | 17X | *PyGFP* | Intact parasites | constitutive GFP; fertile gametes | [3] |
|  |  | *Py17X* | Intact parasites | wild-type; fertile gametes |  |
|  | 33X | *PyWT* | Knockout parasites | wild-type; fertile gametes |  |

Table S2. Number of independent samples (cultures) for mixed-species and control treatments. We set up mixed-species mating cultures from multiple pairs of infections (replicates; n). Several types of control mating culture (i-iii) were required for each experiment (described in the main text). Each infection only contributed to one culture within each treatment or control condition. The analyses in the main text consider whether mating between con- and heterospecifics is random, by comparing the proportion of heterospecific females (*expected hybridization*) to the proportion of hybrid ookinetes (*observed hybridization*). Thus, here we give the mean±SE density of ookinetes observed for each type of mating or control condition.

| **experiment** | **culture type** | **line^1^** | **n (all)** | **n (analysed)^2^** | **Ookinetes per ml** |
| --- | --- | --- | --- | --- | --- |
| Intact parasites | Mixed-species | *PyGFP* | 22 | 20 | Hybrids: 3.8x10^5^±1.4x10^5^  Self: 3.4x10^6^±1x10^6^ |
|  |  | *PbRFP* | 23 | 15 | Hybrids: 2.9x10^5^±1.6x10^5^  Self: 1x10^7^±1.7x10^6^ |
|  | Control (i) | *PbRFP* | 24 | 23 | 2x10^7^±2.3x10^6^ |
|  |  | *PyGFP* | 24 | 23 | 3.7x10^6^±1x10^6^ |
|  | Control (ii) | *PbRFP* | 24 | 24 | 1.1x10^5^±3.4x10^4^ |
|  |  | *PyGFP* | 24 | 24 | 1.5x10^5^±6.2x10^4^ |
|  | Control (iii) | *PbRFP* | 3 | 3 | 3.5x10^6^±2.8 x10^6^ |
|  |  | *PyGFP* | 3 | 3 | 4.9x10^5^±3.1x10^5^ |
| Knockout parasites | Mixed-species | *PbΔp48/45* | 20 | 14 | Hybrids: 8.9x10^4^±1.9x10^4^  Self: 3.3x10^5^±1.4x10^5^ |
|  |  | *PbΔp230* | 17 | 11 | Hybrids: 1.9x10^5^±4.1x10^4^  Self: 3.3x10^5^±1.7x10^5^ |
|  | Control (i) | *PbWT* | 13 | 13 | 3.3x10^6^±1x10^6^ |
|  |  | *PyWT* | 18 | 14 | 9.6x10^5^±2.2x10^5^ |
|  |  | *PbΔp48/45* | 18 | 18 | No ookinetes observed |
|  |  | *PbΔp230* | 15 | 15 | No ookinetes observed |
|  | Control (ii) | *PbΔp48/45* | 5 | 5 | WT: 6.8x10^5^±8.9x10^2^  KO:1.5x10^6^±2.0x10^3^ |
|  |  | *PbΔp230* | 5 | 5 | WT: 7.7x10^5^±1x10^3^  KO:1.3x10^6^±3.7x10^3^ |

^1^ For the mixed-species cultures, this indicates the line donating only females into culture (i.e. for which hybridization is being tested)

^2^ For the experiment using intact parasites, cultures were excluded from analyses if the number of ‘hybrid’ ookinetes present in mixed-species cultures subtracted from the number of ookinetes in control (ii) resulted in negative counts (our results are qualitatively similar if ookinete counts in those cultures are included). For the experiment using knockout parasites, cultures were excluded if *PyWT* did not produce ookinetes from conspecific fertilisations in control (i) or in mixed-species cultures.

**Data analysis**

*Data analysis for results section ‘b) Reproductive interference’*

To test whether reproductive interference exists we first multiplied the number of ookinetes obtained in mixed-species cultures by 1.25 because single-species cultures contained 75 μl of blood but, in mixed-species cultures, only 60 μl of the species donating viable male and female gametes was added. We used linear mixed-effects models (with infection pair as a random effect) to test whether the proportion of conspecific females that were fertilized was influenced by the presence of heterospecifics. We minimised models using log-likelihood ratio tests.

### Data analysis for results section ‘d) Mating is non-random in the absence of P230 and P48/45’

To examine how density-dependent processes and the absence of P230 or P48/45 from females influence hybridisation rates, we used a quasi-binomial generalized linear model. We tested whether *observed hybridisation* was differentially affected by the *P. berghei* line (*PbΔp48/45* or *PbΔp230*), the density of con- and heterospecific females, and interactions between these variables. We minimised models following stepwise deletion of the least significant term and used F-ratio tests to evaluate the change in model deviance until only significant terms remained [14].

We obtained a minimal model, which included the density of con- and hetero-specific females and their interaction. We fitted this model in a generalized linear mixed-effects framework with infection pair as a random effect. To visualize the effects pretdicted by the model, we created a square-matrix of values (500x500) for the densities of con- and heterospecifics by generating two sequences of 500 values (equally-spaced), between the minimum and maximum densities of con- and heterospecific females observed in the experiment. We then inputted this matrix into the minimal GLM to predict how the level of *observed hybridization* correlates with the densities of con- and heterospecific females (Figure 5).

**Molecular Evolution**

*DNA alignment, recombination tests and single locus statistics*

We aligned sequences with ClustalW (in BioEdit; [15]), with adjustments by eye and used GARD [16, 17] in Datamonkey [18, 19] to test for recombination at each locus. We found no statistical support for recombination, consistent with low effective recombination rates and the short gene lengths.

We used DNAsp v5 [20] to compute *D_N_*, *D_S_*, *P_N_*, *P_S_*, *K_A_*, *K_S_*, *K_A_/K_S_*, *π_A_*, *π_S_*, *π_A_/π_S_*, Tajima’s D [21] and single-locus McDonald-Kreitman tests [22]. We measured polymorphism for the following groups*: P. chabaudi chabaudi*, *P. vinckei* unnamed subsp., *P. yoelii yoelii* (subspecies), *P. chabaudi*, *P. yoelii* (species) and *P. vinckei* unnamed subsp.-*P. vinckei petteri*. The latter group was used because this is the only *P. vinckei* subspecies that diverged within the same time frame as the *P. chabaudi* and *P. yoelii* subspecies (others are much older; see [6]). We performed this analysis both at the species and at the subspecies level because our previous work suggests that, for rodent malarias, estimations of polymorphism at the species level can often be inflated [6]. We estimated divergence from an inferred ancestral sequence (as in [6]), for the *P. berghei*-*P. yoelii* and *P. chabaudi*-*P. vinckei* nodes (we did not use *P. berghei* as an intraspecific group due to its extremely low polymorphism; [6]). Furthermore, using the polymorphism and divergence counts above, we used maximum likelihood multi-locus versions of both MK [23] and Hudson-Kreitman-Aguade tests (HKA; [24]), which allow for the comparison of selective patterns in different classes of loci, thus permitting us to compare selection on ‘mating’ and ‘control’ loci (counts for the control loci were obtained from [6]).

*Multi-locus MK and HKA tests*

The original MK test [25] infers positive selection if *D_N_* is larger than would be expected under a model comprising only neutral and constrained mutations. The data used to compute MK tests (*D_N_, D_S_*, *P_N_, P_S_*) can also be used to estimate *α* (the inferred proportion of adaptive non-synonymous substitutions; [26]). For the multi-locus MK test [23], we used three models in which: (i) *α* is constrained to zero at all loci, i.e. no adaptive evolution; (ii) *α* is a free parameter common to all loci; or (iii) *α* is free to differ between mating and control loci. Comparing these models, allows us to test whether positive selection exists and if there is a difference between mating and control loci. We obtained estimates of *α* under model (iii), for mating and control loci and compared models using Akaike weights following ([27]; Akaike weights were calculated from AICc, and can be seen as the weight of evidence in favour of a model, given the relative support for all other models in that model set).

The HKA test detects unusually high or low levels of diversity, given the observed level of divergence between different taxa. This can be used to infer the action of balancing selection where this has resulted in elevated diversity, or selective sweeps where this has resulted in a local decrease in genetic diversity [25]. For the multi-locus HKA test [24], we compared models in which there is no selection at both mating and control loci to models in which selection is allowed to occur at mating loci, and significance was assessed with likelihood ratio tests. We computed the multi-locus MK and HKA tests using the data for all control loci and either all, or each, of the mating loci.

*Codon evolution models*

We fitted two sets of nested codon evolution models (PAML; [28]) to the entire sequence of each mating locus. The different models either constrain all codons to be evolving at a neutral or constrained ratio of non-synonymous to synonymous divergence (*K_A_/K_S_* ≤1; M1a, M7 and M8a) or allow for a proportion of codons to be evolving under positive selection (*K_A_/K_S_* >1; M2a and M8). We compared models using likelihood ratio tests between M8 and M7 (or M8a) and between M2a and M1a. When M2a or M8 fitted the data significantly better, indicating the presence of positive selection, we used a Bayes Empirical Bayes approach to identify specific codons under selection.

**Table S3. Primers Used to Sequence P230, P47 and P48/45.** Cycling conditions for Touch-Down PCRs: (i) 95ºC for 3 min.; (ii) 10 cycles of: 94ºC for 30s, 57ºC for 30s (-1ºC per cycle), 72ºC for 1.5 min.; (iii) 35 cycles of: 94ºC for 30s, 47ºC for 30s, 72ºC for 1.5 min.; (iv) 72ºC for 3 min. If PCR products were negative in gel electrophoresis under these cycling conditions, we carried out new PCR reactions in which the temperatures in step 2 of (ii) and (iii) were increased or decreased by 5ºC. Primers for: **P. yoelii*; ***P. chabaudi* and *P. berghei*; ****P. chabaudi* and *P. yoelii*.

| **species** | **locus** | **forward** | **reverse** | **internal** |
| --- | --- | --- | --- | --- |
| universal | P230 region I | GTAGATATMTCATTTCAAGTACCAGC | TTCAGGTGGRCATTCAAAAGCTAT | TCCACATTCAGAAATACTTGTAGA |
|  | P230 region II | CAATCAACTACAAACTGATGGT | GAATATTTTCCTGTAAAATCACAACCA | GGAAGGAATGGAAACTAATGTCT |
|  | P47 | ACGTCGTTCTGTTTTCTRTATC | CAAATCCGAATACAGAAATAATTGCATG; | - |
|  |  |  | TTCTATTATGGCTTGTAACGAATATTCAA |  |
|  | P48/45 | CACACACACTTTCTCGCACATC; | - | - |
|  |  | CGCACATCAAAGTTATCAARTGC |  |  |
| *P. berghei* and  *P. yoelii* | P230 region I | GTATTATCTATGAATACAGAAATAGAGTCT | TTGATTGTTTCAGGTGGGC | AACAGATTCTATTCGTATGCTCAATTA |
|  | P230 region II | GTAAAATTACAATCAGCTTTACCTGGAG | CATGCAAATCCCGCAAATTTGTTATTGG; | *****TCGATGATATATTACCAGGAGCTATC |
|  |  |  | GGATTTGCATGTCCAAGTAAC |  |
|  | P47 | CTAAAGACACCTCACACATTTTCGTTG; | GCTCTCAAAAGCTTTGTTCATTGCTC; | - |
|  |  | TGCCCATCTTTTTCATAACTATGC | GAGCAATGAACAAAGCTTTTGAG |  |
|  | P48/45 | - | CACAGAAATAATTGCATGTACACGTTC | CATGTACTAATGCACTTCTTCCATC; |
|  |  |  |  | GAACAAACAGGATTAATAGAATATACACTTG |

**Table S3 (continued).**

| **species** | **locus** | **forward** | **reverse** | **internal** |
| --- | --- | --- | --- | --- |
| *P. chabaudi* | P230 region I | ATTACCTATGAATACAGAAATTGAATCCA | TTGACAGTTTCAGGTGGAC | TGCTCAATTAGCATGTCCAAG |
|  | P230 region II | GTAAAATTACAAACTGCATTACCTGGAC | CAATATAATTACTTGGGCATGCAAATCCA; | ******TTGATGATATATTACCAGGAGCTATC |
|  |  |  | GGATTTGCATGCCCAAGTAAT |  |
|  | P47 | CTCGAAACAATTCACATTTTTGCGTTG | GTTGACTCTGAAAATCTTTGTTCATTACTC | - |
|  | P48/45 | - | - | *******GCACTAATGCACTTCTTCCATC; |
|  |  |  |  | GAAAAAACAGGATTAGTAGAATATACACTTG |

**SUPPLEMENTARY RESULTS**

### Hybridisation can occur between P. berghei and P. yoelii: controls

To validate the results from the mixed-species cultures of intact parasites, we performed a series of control cultures, and used the resulting ookinete densities (Table S2) to verify the assumptions that: (i) Conspecific matings occur readily in cultures containing only *PbRFP* or *PyGFP* and we observed no significant difference between the proportion of fertilised females for either species (Likelihood ratio test [LRT]: *χ^2^_1_* = 0.448; *p* = 0.503). (ii) There was no significant difference between untreated and aphidocolin treated-females in their fertilisation rates by conspecific males, for either species (*PbRFP*: *t* = 3.63, *df* = 2, *p* = 0.0682; *PyGFP*: *t* = 2.208, *df* = 2, *p* = 0.158). (iii) Aphidicolin blocks (almost completely) the formation of fertile male gametes. Mating success of aphidocolin treated males was reduced by 99.4% for *PbRFP* (95% confidence interval [CI]: 99.0 – 99.8) and by 95.7% for *PyGFP* (CI: 90.2 – 100) compared to untreated males. The rare production of fertile males after aphidicolin treatment did not result in a significant difference in the proportion of fertilised females for each parasite species (LRT χ^2^_1_ = 2.12; *p* = 0.146). Therefore, the values of *observed hybridisation* presented for the mixed-species mating cultures (Figure 2), have been corrected for the potential presence of fertile males after aphidicolin-treatment. We did this by subtracting the number of ookinetes/ml produced by aphidicolin-treated males in these control cultures from the number of hybrid ookinetes observed in the mixed-species mating cultures. We were able to calculate a specific correction for each individual mixed-species mating culture because each replicate pair of mixed-species infections was also examined in these control cultures.

**P230 and P48/45 are pre-zygotic reproductive barriers: controls**

To validate the results from the mixed-species cultures of knockout parasites, we set up a series control cultures, and used the resulting ookinete densities (Table S2) to verify the assumptions that: (i) The mutant lines *PbΔp230* and *PbΔp48/45* do not produce ookinetes when cultured alone, and that *PbWT* and *PyWT* readily produced ookinetes, with no significant difference between species in the proportion of fertilised females (*W* = 86.5, *p* = 0.8461). (ii) Female gametes of the mutant lines *PbΔp230* and *PbΔp48/45* are fertile and there was no significant difference between *PbΔp230* (*t* = 1.2578, *df* = 4, *p* = 0.2769) or *PbΔp48/45* (*t* = -1.2291, *df* = 4, *p* = 0.2864) in the proportion of wild-type or mutant females fertilised by wild-type males.

**Table S5. Multi-Locus MK Tests for the Subspecies.** This analysis was carried using all control loci and either all or each of the mating loci separately. The Akaike weight for model (iii) (allows *α* to vary between mating and control loci) is given and highlighted in bold when this is the best model. Akaike weights are calculated for the set of models tested and always add up to 1. *α* and standard error was calculated from 1000 permutations on the results of model (iii).

| **locus** | **class** | ***P. c. chabaudi*** | | ***P. v.* subsp.** | | ***P. y. yoelii*** | |
| --- | --- | --- | --- | --- | --- | --- | --- |
|  |  | **Akaike weight** | **α (std. error)** | **Akaike weight** | **α (std.**  **error)** | **Akaike weight** | **α (std. error)** |
| ***all*** | mating | **0.99** | 0.72 (0.004) | **0.99** | 0.61 (0.004) | **0.99** | 0.38 (0.011) |
|  | control |  | 0.32 (0.006) |  | -0.041 (0.008) |  | -1.01 (0.019) |
| ***P230 region I*** | mating | **0.99** | 0.85 (0.001) | **0.99** | 0.81 (0.002) | **0.99** | 0.71 (0.003) |
|  | control |  | 0.44 (0.005) |  | 0.29 (0.004) |  | -0.60 (0.016) |
| ***P230 region II*** | mating | 0.29 | 0.64 (0.004) | 0.28 | 0.55 (0.004) | 0.14 | -0.93 (0.016) |
|  | control |  | 0.43 (0.005) |  | -0.026 (0.005) |  | -1.07 (0.019) |
| ***P47*** | mating | **0.99** | 0.82 (0.002) | **0.76** | 0.66 (0.003) | **0.99** | 0.72 (0.003) |
|  | control |  | 0.33 (0.006) |  | 0.081 (0.005) |  | -0.62 (0.017) |
| ***P48/45*** | mating | 0.15 | 0.42 (0.007) | 0.48 | 0.67 (0.003) | 0.18 | 0.19 (0.010) |
|  | control |  | 0.43 (0.006) |  | 0.26 (0.004) |  | -0.45 (0.016) |

* For a given subspecies, the values of *α* for the control loci change with the mating locus used, even though the data for the control loci was the same. This is because the likelihood models include the following parameters that we assume to be constant across all loci: neutral diversity and divergence per site, and selective constraint.

**Table S6. Multi-Locus MK Tests for the Species.** This analysis was carried using all control loci and either all or each of the mating loci separately. The Akaike weight for model (iii) (allows α to vary between mating and control loci) is given and highlighted in bold when this is the best model. Akaike weights are calculated for the set of models tested and always add up to 1. α and standard error was calculated from 1000 permutations on the results of model (iii).

| **locus** | **class** | ***P. chabaudi*** | | ***P. v.* subsp. – *P. v. petteri*** | | ***P. yoelii*** | |
| --- | --- | --- | --- | --- | --- | --- | --- |
|  |  | **Akaike weight** | **α (std. error)** | **Akaike weight** | **α (std. error)** | **Akaike weight** | **α (std. error)** |
| ***all*** | mating | **0.98** | 0.63 (0.004) | **0.99** | 0.70 (0.003) | **0.99** | 0.37 (0.007) |
|  | control |  | 0.25 (0.007) |  | 0.10 (0.007) |  | -1.53 (0.028) |
| ***P230 region I*** | mating | **0.99** | 0.85 (0.002) | **0.99** | 0.85 (0.001) | **0.99** | 0.74 (0.003) |
|  | control |  | 0.46 (0.004) |  | 0.37 (0.004) |  | -0.71 (0.016) |
| ***P230 region II*** | mating | 0.3 | 0.66 (0.004) | 0.45 | 0.68 (0.003) | 0.14 | -0.50 (0.019) |
|  | control |  | 0.48 (0.005) |  | 0.19 (0.005) |  | -0.61 (0.016) |
| ***P47*** | mating | **0.98** | 0.73 (0.003) | **0.9** | 0.74 (0.002) | **0.99** | 0.50 (0.006) |
|  | control |  | 0.24 (0.007) |  | 0.21 (0.005) |  | -1.75 (0.030) |
| ***P48/45*** | mating | 0.21 | 0.36 (0.009) | **0.68** | 0.74 (0.003) | 0.42 | 0.26 (0.009) |
|  | control |  | 0.50 (0.005) |  | 0.38 (0.004) |  | -0.70 (0.016) |

* For a given species, the values of *α* for the control loci change with the mating locus used, even though the data for the control loci was the same. This is because the likelihood models include the following parameters that we assume to be constant across all loci: neutral diversity and divergence per site, and selective constraint.

**Table S7. Codon Evolution Models and Likelihood Ratio Tests.** Likelihood ratio tests comparing nested models of codon evolution, estimates of *K_A_/K_S_* values and the % codons under positive selection (*K_A_/K_S_* >1) for each locus. M1a, M7 and M8a assume neutral evolution (*K_A_/K_S_* ≤1) and models M2a and M8 assume there is a class of codons under positive (*K_A_/K_S_* >1). *p* < 0.05 indicate that positive selection was detected.

| **locus** | **model comparison** | **likelihood ratios and *p*-values** | **no. of codons analyzed** | **% selected codons and *K_A_/K_S_* of selected class for M2a or M8** |
| --- | --- | --- | --- | --- |
| ***P230 region I*** | M1a vs. M2a | χ^2^_2_= 0; *p*=1 | 409 | - |
|  | M7 vs. M8 | χ^2^_2_=13.11; *p*=0.0014 |  | 3.5%; *K_A_/K_S_*=2.38 |
|  | M8a vs. M8 | χ^2^_1_=3.16; *p*=0.038 |  |  |
| ***P230 region II*** | M1a vs. M2a | χ^2^_2_=0; *p*=1 | 365 | - |
|  | M7 vs. M8 | χ^2^_2_=10.62; *p*=0.005 |  | 1.5%; *K_A_/K_S_*=3.83 |
|  | M8a vs. M8 | χ^2^_1_=5.79; *p*=0.008 |  |  |
| ***P47*** | M1a vs. M2a | χ^2^_2_=8.65; *p*=0.013 | 428 | 5.9%; *K_A_/K_S_*=2.71 |
|  | M7 vs. M8 | χ^2^_2_=13.67; *p*=0.001 |  | 9.3%; *K_A_/K_S_*=2.37 |
|  | M8a vs. M8 | χ^2^_1_= 9.74; *p*=0.001 |  |  |
| ***P48/45*** | M1a vs. M2a | χ^2^_2_=4.58; *p*=0.10 | 478 | 1.9%; *K_A_/K_S_*=3.67 |
|  | M7 vs. M8 | χ^2^_2_=9.04; *p*=0.011 |  | 3.1%; *K_A_/K_S_*=3.17 |
|  | M8a vs. M8 | χ^2^_1_=5.44; *p*=0.01 |  |  |

**Table S8. Codons with *K_A_/K_S_* > 1, as Identified by Bayes Empirical Bayes (BEB) Analysis.** Codons in bold were detected by BEB for both M8 and M2a. Labelled codons have a posterior probability *p* > 0.90 (#) or *p* > 0.95 (*) or have been previously suggested to be under positive selection (§) in [5]. As only a small number of individual codons show high support for positive selection (posterior probability >0.9), these inferences should be treated with care. Codons in which ? replaces the single letter amino acid code correspond to missing data in the sequence used by PAML as reference for codon identity.

| **locus** | **codons with *K_A_*/*K_S_*>1** |
| --- | --- |
| ***P230 region I*** | 70T, 92F, 95S, 98V(§), 113D(#), 144N(#), 167Q, 171V, 202S, 254N, 340S, 381? |
| ***P230 region II*** | 25?(#), 27?, 82L, 89V, 127L(#), 139L, 173K, 259L, 336?(#) |
| ***P47*** | **9?(§)**, 12?, 27F(§), **32V(§)**, 69I, 71L, 79N(§), 82E(§), 84M, 125S, 155R(§), **158I(*)**, 162G, **163E(§)**, **165I(§)**, 182Q, 186Q(§), **195T(*)**, 213V, 219G, 243L, 275K, 277K, 289V, **293K**, **324A**, 350N, 354V, 387I, **400H**, **404K**, 428? |
| ***P48/45*** | **12?, 30?, 111I, 139K, 140T(#, §), 164S, 204T, 220Q, 230N, 233D(§), 361S(*, §), 416I** |

**SUPPLEMENTARY LEGENDS**

**Table S4. Polymorphism, divergence and single locus MK tests and Tajima's D (silent sites).** MK - McDonald-Kreitman; n - number of genotypes; *P_s_*, *P_n_* - synonymous and non-synonymous polymorphisms, *D_s_*, *D_n_* - synonymous and non-synonymous divergence; *π_A_*, *π_S_* - non-synonymous and synonymous polymorphism rate; *K_A_*, *K_S_* - non-synonymous and synonymous divergence rate; Statistical significance: # 0.05 < *p <* 0.10; * *p* < 0.05; ** *p* < 0.01.

**SUPPLEMENTARY REFERENCES**

1. Guttery D.S., Holder A.A., Tewari R. 2012 Sexual Development in Plasmodium: Lessons from Functional Analyses. *Plos Pathog* **8**(1). (doi:Artn E1002404

Doi 10.1371/Journal.Ppat.1002404).

2. Liu Y.J., Tewari R., Ning J., Blagborough A.M., Garbom S., Pei J.M., Grishin N.V., Steele R.E., Sinden R.E., Snell W.J., et al. 2008 The conserved plant sterility gene HAP2 functions after attachment of fusogenic membranes in Chlamydomonas and Plasmodium gametes. *Gene Dev* **22**(8), 1051-1068. (doi:Doi 10.1101/Gad.1656508).

3. Ono T., Tadakuma T., Rodriguez A. 2007 Plasmodium yoelii yoelii 17XNL constitutively expressing GFP throughout the life cycle. *Exp Parasitol* **115**(3), 310-313. (doi:10.1016/j.exppara.2006.09.008).

4. van Dijk M.R., Janse C.J., Thompson J., Waters A.P., Braks J.A.M., Dodemont H.J., Stunnenberg H.G., van Gemert G.J., Sauerwein R.W., Eling W. 2001 A central role for P48/45 in malaria parasite male gamete fertility. *Cell* **104**(1), 153-164.

5. van Dijk M.R., van Schaijk B.C.L., Khan S.M., van Dooren M.W., Ramesar J., Kaczanowski S., van Gemert G.-J., Kroeze H., Stunnenberg H.G., Eling W.M., et al. 2010 Three members of the 6-cys protein family of *Plasmodium* play a role in gamete fertility. *PLoS Pathog* **6**(4), e1000853. (doi:doi:10.1371/journal.ppat.1000853).

6. Ramiro R., Reece S., Obbard D. 2012 Molecular evolution and phylogenetics of rodent malaria parasites. *BMC Evol Biol* **12**(1), 219.

7. Pacheco M.A., Battistuzzi F.U., Junge R.E., Cornejo O.E., Williams C.V., Landau I., Rabetafika L., Snounou G., Jones-Engel L., Escalante A.A. 2011 Timing the origin of human malarias: the lemur puzzle. *BMC Evol Biol* **11**(1), 299. (doi:10.1186/1471-2148-11-299).

8. Silva J.C., Egan A., Arze C., Spouge J.L., Harris D.G. 2015 A new method for estimating species age supports the co-existence of malaria parasites and their mammalian hosts. *Molecular Biology and Evolution*. (doi:10.1093/molbev/msv005).

9. Wéry M. 1966 Etude du cycle de Plasmodium berghei yoelii en vue de la production massive de sporozoites viables et de formes exoerythrocytaires. *Ann Soc Belge Méd Trop* **46**, 755-788.

10. Chance M.L., Momen H., Warhurst D.C., Peters W. 1978 The chemotherapy of rodent malaria, XXIX DNA relationships within the subgenus Plasmodium (Vinckeia). *Annals of Tropical Medicine and Parasitology* **72**(1), 13.

11. Peters W., Chance M.L., Lissner R., Momen H., Warhurst D.C. 1978 The chemotherapy of rodent malaria, XXX. The enigmas of the'NS lines' of P. berghei. *Annals of Tropical Medicine and Parasitology* **72**(1), 23.

12. Killick-Kendrick R., Peters W. 1978 *Rodent Malaria*, Academic Press.

13. Ponzi M., Siden-Kiamos I., Bertuccini L., Curra C., Kroeze H., Camarda G., Pace T., Franke-Fayard B., Laurentino E.C., Louis C., et al. 2009 Egress of *Plasmodium berghei* gametes from their host erythrocyte is mediated by the MDV-1/PEG3 protein. *Cell Microbiol* **11**(8), 1272-1288. (doi:Doi 10.1111/J.1462-5822.2009.01331.X).

14. Zuur A.F., Ieno E.N., Walker N., Saveliev A.A., Smith G.M. 2009 *Mixed Effects Models and Extensions in Ecology with R*. 1st ed. New York, Springer.

15. Hall T.A. 1999 BioEdit: a user-friendly biological sequence alignment editor and analysis program for Windows 95/98/NT. *Nucleic Acids Symposium Series* **41**(41), 95-98.

16. Kosakovsky Pond S., Posada D., Gravenor M., Woelk C., Frost S. 2006 Automated phylogenetic detection of recombination using a genetic algorithm. *Mol Biol Evol* **23**(10), 1891.

17. Kosakovsky Pond S., Posada D., Gravenor M., Woelk C., Frost S. 2006 GARD: a genetic algorithm for recombination detection. *Bioinformatics* **22**(24), 3096.

18. Delport W., Poon A.F.Y., Frost S.D.W., Kosakovsky Pond S.L. 2010 Datamonkey 2010: a suite of phylogenetic analysis tools for evolutionary biology. *Bioinformatics* **26**(19), 2455-2457. (doi:10.1093/bioinformatics/btq429).

19. Pond S.L.K., Frost S.D.W. 2005 Datamonkey: rapid detection of selective pressure on individual sites of codon alignments. *Bioinformatics* **21**(10), 2531-2533. (doi:10.1093/bioinformatics/bti320).

20. Librado P., Rozas J. 2009 DnaSP v5: a software for comprehensive analysis of DNA polymorphism data. *Bioinformatics* **25**(11), 1451-1452. (doi:10.1093/bioinformatics/btp187).

21. Tajima F. 1989 Statistical method for testing the neutral mutation hypothesis by DNA polymorphism. *Genetics* **123**(3), 585-595.

22. McDonald J.H., Kreitman M. 1991 Adaptive protein evolution at the Adh locus in *Drosophila*. *Nature* **351**(6328), 652-654. (doi:10.1038/351652a0).

23. Welch J.J. 2006 Estimating the Genomewide Rate of Adaptive Protein Evolution in Drosophila. *Genetics* **173**(2), 821-837. (doi:10.1534/genetics.106.056911).

24. Wright S.I., Charlesworth B. 2004 The HKA Test Revisited: A Maximum-Likelihood-Ratio Test of the Standard Neutral Model. *Genetics* **168**(2), 1071-1076. (doi:10.1534/genetics.104.026500).

25. Kreitman M. 2000 Methods to detect selection in populations with applications to the Human. *Annu Rev Genomics Hum Genet* **1**(1), 539-559. (doi:doi:10.1146/annurev.genom.1.1.539).

26. Eyre-walker A. 2006 The genomic rate of adaptive evolution. *Trends Ecol Evol* **21**(10), 569-575. (doi:10.1016/j.tree.2006.06.015).

27. Burnham K.P., Anderson D.R. 2002 *Model Selection and Multimodel Inference: A Practical Information-Theoretic Approach*, Springer.

28. Yang Z.Z. 2007 PAML 4: phylogenetic analysis by maximum likelihood. *Mol Biol Evol* **24**(8), 1586-1591. (doi:10.1093/molbev/msm088).
